# Assessing the wellbeing of Chinese university students: validation of a Chinese version of the college student subjective wellbeing questionnaire

**DOI:** 10.1186/s40359-021-00569-8

**Published:** 2021-05-01

**Authors:** Yan Zhang, Richard Carciofo

**Affiliations:** Department of Health and Environmental Sciences, Xi’an Jiaotong-Liverpool University, 111 Ren’ai Road, Suzhou, 215123 China

**Keywords:** Chinese students, Student wellbeing, Questionnaire validation, Determinants of wellbeing, Positive psychology, Subjective happiness

## Abstract

**Background:**

In response to the rising concern with promoting the wellbeing of university students and relative lack of domain-specific wellbeing measurement instruments in China, the current study aimed to validate a Chinese version of the College Student Subjective Wellbeing Questionnaire (CSSWQ), a 16-item self-report English-language rating scale assessing four aspects of wellbeing (academic satisfaction, academic efficacy, school connectedness, and college gratitude).

**Methods:**

The Chinese translation of the CSSWQ, the Students’ Life Satisfaction Scale, the Positive and Negative Affect Schedule, the 10-Item Big Five Personality Inventory, and demographic questions were completed by 252 Chinese students at a university in Suzhou, China.

**Results:**

Exploratory factor analysis found four factors each with the same four items as in the original English scale. Each subscale showed good internal consistency. Test–retest for a one-month interval showed generally moderate reliability. As predicted, Pearson correlational analysis found positive correlations between the Chinese CSSWQ and life satisfaction, positive affect, extraversion, and GPA, and negative correlations with neuroticism and negative affect. Monthly income had small negative correlations with academic satisfaction and academic efficacy, smoking had a small positive correlation with school connectedness, and exercise had a small positive correlation with academic efficacy.

**Conclusion:**

Data for the Chinese CSSWQ in the current study showed validity and reliability, supporting the use of this instrument as a measurement of college student wellbeing in China.

## Introduction

Mental health problems are an increasing global burden. Using disability adjusted life years (DALYs) as measurement of the burden of disease, in 1990, all mental and behavioural disorders accounted for 5.7% of the global DALYs, while in 2010, their share increased up to 7.4% of the global DALYs [36]. This increase may be due to the diagnosis of newly classified disorders such as childhood disorders and eating disorders, together with historically underestimated disorders such as major depressive disorder [36], and also related to societal transitions as people today face a wider variety of challenges compared to those in the past [21].

For adolescents and young adults aged 15–39, mental and behavioural disorders are one of the main causes of DALYs [36], and attendance at college/university may have an influence on wellbeing. Bewick, Koutsopoulou, Miles, Slaa, and Barkham [3] found that students experience lifetime highest levels of distress after they register at university, which peaks during the first semester and remains stably higher than pre-university levels. College time predominantly features anxiety, while depressive symptoms are less dominant in the beginning and reach their zenith during the final year of study [1, 3, 7]. Major life transitions (e.g., first time far from home, job seeking), social challenges (e.g., making friends), and academic challenges (e.g., different teaching/learning styles) may be some influencing factors [28, 40, 48].

Students with mental illnesses show less engagement in class and poorer social relationships, which are associated with lower graduation rates [48]. Typically, depression relates to students’ poorer sleep quality, less willingness to do physical activities, higher chance of alcohol abuse, and increased rates of daily cigarette smoking [5, 14, 54], which would increase the risk of cardiovascular diseases, cancer, neurological diseases, etc. [10, 41]. Likewise, the physical health consequences may in turn aggravate one’s psychological problems. Due to such severe potential outcomes, monitoring students’ wellbeing and developing suitable interventions should be included in university planning [30, 43], which should consider external factors (e. g., financial status, social support, and professional help) and internal factors (e. g., personality) [20, 24, 31]. For instance, extraversion predicts more wellbeing [31], and neuroticism predicts less wellbeing [12, 26].

In recent years, with increased concern with promoting mental health, more emphasis has been put on positive aspects of psychology [16]. Commonly used questionnaires that investigate people’s wellbeing, focusing on positive feelings and experiences, include the Warwick-Edinburgh Mental Wellbeing Scale (WEMWBS) [52], the Subjective Happiness Scale (SHS) [32], the Personal Growth Initiative Scale (PGIS) [47], and the Students’ Life Satisfaction Scale (SLSS) [23]. Similar to the function of the WEMWBS described by Tennant et al. [52], all of these scales can be useful for monitoring the wellbeing of groups, investigating determinants of wellbeing at the population level, evaluating programmes and projects which potentially affect wellbeing, and enabling self-reflection before health interventions.

However, there has also been an increasing focus on developing domain-specific measures of wellbeing, rather than relying on more domain-general measures. For measuring the wellbeing of college students, researchers in the US [45] developed and validated a 15-item, four-subscale self-report rating questionnaire, the College Student Subjective Wellbeing Questionnaire (CSSWQ). Renshaw [44] later developed a 16-item version of the CSSWQ, which includes four categories (academic satisfaction, academic efficacy, school connectedness, and college gratitude) in four subscales, which, through wording adaptation, were developed from general life satisfaction, self-efficacy, gratitude, and social connectedness scales. Renshaw [44] found strong convergent validity with several other domain-general measurements, including the Positive and Negative Affect Schedule (PANAS), and several additional validity measures, including grade point average (GPA).

The CSSWQ could be a useful instrument in China as well if validated in the Chinese context. Although Chinese researchers have translated and validated several scales related to wellbeing, including the Chinese WEMWBS [11], SHS [37], PGIS [55], SLSS [25], and PANAS [22], none focuses specifically on college students’ wellbeing.

Therefore, the aim of the present study was to translate and validate a Chinese version of the CSSWQ. Validation involved factor analysis of the scale structure, and correlations with theoretically related variables. Specifically, it was predicted that CSSWQ scores would correlate positively with life satisfaction, positive affect, extraversion and GPA, and negatively with negative affect and neuroticism. Reliability was tested by measures of internal consistency and test–retest reliability. In addition, associations between CSSWQ scores and agreeableness, openness, and conscientiousness were also explored, as were associations with exercise, alcohol drinking, cigarette smoking, and money received monthly from family.

## Methodology

### Sample

Students were recruited at a university in Suzhou, China by convenience sampling (N = 252, year 1 = 48.0%, year 2 = 16.7%, year 3 = 21.4%, year 4 = 12.7%, Master = 1.2%); 171 females (67.9%) and 81 males (32.1%). Inclusion criteria were being a Chinese student of the university, aged at least 18 years. The age range of the participants was from 18 to 27 (mean = 19.49, SD = 1.449). The average age was 19.63 (SD = 1.771) for males and 19.42 (SD = 1.269) for females (t = 0.951, p = 0.344). The sample size was adequate to establish moderate-size correlations with 80% power at the 5% significance level [6].

### Materials

The cross-sectional survey consisted of the following Chinese-language questionnaires. Considering that the topic of an earlier questionnaire might influence/bias how participants respond to a later one [29], all four questionnaires were counter-balanced and 24 different versions of the survey were utilised to give some variation and reduce order effects.

1. *The College Student Subjective Wellbeing Questionnaire (CSSWQ) *[44, 45]
  This has 16 items, each scored from 1 (strongly disagree) to 7 (strongly agree). The translation of the CSSWQ into Chinese was done by a native Chinese who speaks proficient English. Considering that a matter may be expressed differently in different cultures, several wordings were slightly adjusted for better understanding in Chinese sentences while retaining the original meaning, e. g., item 5 replaced “I am a hard worker” in English with “I work hard” in Chinese, and item 12 replaced “like me the way I am” with “like the real me”. Back translation was then done by another native Chinese-speaker with high English proficiency, and then examined and verified by a native English-speaker. The result indicated that the Chinese version conveys the same meanings as the original. In addition, pilot tests of the CSSWQ-Chinese were done on native Chinese-speakers who checked the wording of the CSSWQ-Chinese and suggested that the context is meaningful.
2. *The Students' Life Satisfaction Scale (SLSS) *[23]*,Chinese version: *[25]
  The SLSS contains 5 items scored from 1 (strongly disagree) to 6 (strongly agree). The wording of an item which includes reference to “most kids”, was changed from ‘kids’ to ‘people’.
3. *The Positive and Negative Affect Schedule (PANAS) *[53]*,Chinese version: *[22]
  The PANAS consists of 10 items for positive affect and 10 items for negative affect, each scored from 1 (very slightly or not at all) to 5 (extremely).
4. *The 10-Item Big Five Personality Inventory (BFI-10)*[42]*,Chinese version: *[4]
  The BFI-10 consists of two items for each big five dimension (extraversion, agreeableness, conscientiousness, neuroticism, and openness), scored from 1 (disagree strongly) to 5 (agree strongly), with 5 items reverse-scored.
5. *Demographic questions*

These included self-reported GPA, frequency of exercise, frequency of drinking alcohol, frequency of smoking, and money received monthly from family. Response options for GPA ranged from “bad” to “excellent”, scored 1–5. Response options for exercise and drinking ranged from “never” to “almost every day”, scored 1–5. Response options for smoking ranged from “never” to “on average > 10 per day”, scored 1–5. Response options for money ranged from < 500rmb (Chinese Yuan, approximately 76 US dollars) to > 5500rmb (approximately 840 US dollars), in 500rmb intervals, scored from 1 to 16.

### Procedure

Potential participants were approached on the university campus. Paper copies of the questionnaire began with a briefing which explained the aims of the study, and that: participation was voluntary; participation could be withdrawn at any time; the questionnaires would be kept confidential, and the data would be anonymised. Students who agreed to do the retest provided an email address for this purpose, and received a retest email 4 weeks after the first survey. All participants gave signed informed consent, and 71 participants completed the retest. The study protocol was reviewed and approved by the faculty supervisor in accordance with research policies of Xi’an Jiaotong-Liverpool University, and in accordance with the guidelines of the university’s Research Ethics Sub-Committee.

### Data analysis

All questionnaires had complete responses except for one participant missing a response for monthly money received from family, and one participant with multiple responses for this item. These two cases were excluded from analysis for this item. Also, one participant had a single missing response for one of the PANAS items. The participant’s mean score for the corresponding subscale was used as a substitute value for the missing response. Finally, one participant failed to complete any PANAS items (leaving N = 251 for the PANAS). Exploratory factor analysis (EFA) was used to check the structure of the CSSWQ-Chinese, using maximum likelihood estimation and Promax rotation, as used in the original CSSWQ study [45]. Testing new translations of scales with EFA may reveal culture-specific differences in the scale structure, or differences due to the translation process [38]. Descriptive statistics (mean, standard deviation, range, skewness, kurtosis, and Cronbach’s alpha) are provided for each scale and subscale. Pearson correlational analysis was conducted to check the construct validity of the CSSWQ-Chinese by assessing whether the CSSWQ scores correlate with PANAS, SLSS, BFI-10, and GPA as expected. Incremental validity of the CSSWQ in predicting GPA was tested with hierarchical linear regression. Test–retest reliability of CSSWQ scores over four weeks was tested with Pearson correlations; for comparison, intra-class correlations (single-measurement, two-way mixed-effects model, for absolute agreement) were also calculated. Pearson correlations were conducted to explore associations between student wellbeing and other variables including frequency of drinking alcohol, smoking, and exercising, and financial situation. Analysis was conducted using SPSS (version 24).

## Results

### Structural validity

EFA was conducted using maximum likelihood estimation and Promax (oblique) rotation, consistent with the original CSSWQ study [45]. The factorability was supported by the Kaiser–Meyer–Olkin measure of sampling adequacy (0.867), and Bartlett’s test of sphericity (Chi-square = 2828.749, df = 120, p ≤ 0.001), and the anti-image correlations were all > 0.7 [15]. Based on the criteria of eigenvalues greater than 1, four initial factors were extracted explaining 74.636% of the variance. Factor 1 had an initial eigenvalue of 6.857 and explained 42.856% of the total variance, factor 2 had an initial eigenvalue of 2.918 and explained 18.238% of the variance, factor 3 had an initial eigenvalue of 1.115 and explained 6.970% of the variance, and factor 4 had an initial eigenvalue of 1.052 and explained 6.573% of the variance. Factor 5 had an initial eigenvalue of 0.635 and explained 3.969% of the variance.

However, for the four extracted factors the values were: 6.346 (39.661% of variance), 2.701 (16.879% of variance), 0.841 (5.258% of variance), and 0.848 (5.300% of variance), and either two or four factors could be identified from the scree plot. As noted above, factors 1 and 2 explained much more of the variance than factors 3 and 4. So, to rule out one possibility, it was assumed that there were two factors and the number of factors to extract was fixed as two. Under this circumstance, according to the pattern matrix (showing each item’s regression coefficient on each factor) and structure matrix (showing correlations between items and factors) [15], items 2, 3, 4, 5, 6, 7 and 8 had higher loadings on factor 1, while items 1, 9, 10, 11, 12, 13, 14, 15 and 16 had higher loadings on factor 2.

However, reviewing the items did not show a clear way to categorize them into two factors. The structure would involve combining the original factors 1 and 2 (except for item 1), and combining the original factors 3 and 4 (plus item 1). However, the concepts in the original factors 1 and 2 (academic satisfaction and academic efficacy), and in factors 3 and 4 (school connectedness and college gratitude) are distinguishable, and distinguishing them was not problematic in the translation process.

In contrast, the pattern of loadings for four factors was easier to interpret as it exactly matched the revised CSSWQ [44]. In this case, in the pattern matrix (see Table 1), the four factors each involved the same four items as identified in the original study, with all loadings being > 0.4: factor 1 (items 5–8) being academic efficacy, factor 2 (items 13–16) being college gratitude, factor 3 (items 1–4) being academic satisfaction, and factor 4 (items 9–12) being school connectedness [44]. The highest loadings in the structure matrix supported the same four factors as well (see Table 1). However, there were also some high cross-loadings, indicating that the factors are moderately/strongly correlated, which was also found in the original study [44].

**Table 1:** Pattern matrix and structure matrix of the CSSWQ (four factors)

Item	Pattern matrix				Structure matrix			
	Factor				Factor			
	1	2	3	4	1	2	3	4
1	− .147	.121	**.889**	− .086	.376	**.458**	**.813**	**.425**
2	.306	− .026	**.527**	− .050	**.597**	.254	**.668**	.313
3	.339	− .091	**.630**	− .026	**.684**	.258	**.773**	.365
4	.050	− .018	**.770**	.078	**.523**	.397	**.834**	**.502**
5	**.974**	.081	− .078	− .071	**.924**	.201	**.495**	.221
6	**1.035**	.037	− .149	− .031	**.946**	.160	**.462**	.216
7	**.744**	− .073	.058	.092	**.790**	.158	**.512**	.301
8	**.656**	.017	.245	.016	**.809**	.274	**.648**	.353
9	.112	.014	.323	**.419**	**.429**	**.430**	**.624**	**.637**
10	− .125	.195	.080	**.479**	.103	**.483**	.358	**.598**
11	.007	− .053	− .035	**.985**	.267	**.501**	**.482**	**.938**
12	.008	.125	− .107	**.614**	.152	**.431**	.291	**.631**
13	.004	**.608**	.094	.091	.210	**.705**	**.430**	**.494**
14	.060	**.915**	− .042	− .016	.217	**.899**	**.413**	**.508**
15	− .068	**.886**	.179	− .071	.196	**.914**	**.514**	**.518**
16	.068	**.773**	− .137	.134	.184	**.800**	.338	**.526**

Maximum likelihood extraction, with Promax rotation (with Kaiser Normalization); factor loadings > .4 are in bold

### Descriptive statistics

*Table **2* shows the descriptive statistics of the CSSWQ, including skewness and kurtosis. All of the values of skewness and kurtosis were within an acceptable range ( <|1|), indicating approximate normality [35]. The mean of college gratitude was quite high in the scale range compared with other aspects of wellbeing, indicating that for this sample at least, there were generally high scores for gratitude. Cronbach’s alpha coefficients, which were calculated to examine the internal consistency of the CSSWQ-Chinese data, were all > 0.7 and mostly > 0.8, largely consistent with results for the English CSSWQ for which Cronbach’s alphas were no less than 0.79 [44]. The ranges of corrected item-total correlations for each subscale were: academic satisfaction, 0.637 to 0.750, academic efficacy, 0.774 to 0.882; school connectedness, 0.516 to 0.759; college gratitude, 0.661 to 0.843.

**Table 2:** Descriptive statistics for the CSSWQ

CSSWQ	Range (possible)	Mean (SD)	Skewness (SE)	Kurtosis (SE)	Cronbach's alpha	Test–retest Pearson correlation (intra-class correlation with 95% confidence interval)
Academic Satisfaction	8–28 (4–28)	18.45 (4.275)	− .036 (.153)	− .073 (.306)	.854	.746*** (.731; CI = .600 to .824)
Academic Efficacy	4–28 (4–28)	17.52 (4.797)	− .081 (.153)	− .196 (.306)	.922	.695*** (.687; CI = .541 to .792)
School Connectedness	12–28 (4–28)	20.87 (3.680)	− .191 (.153)	− .493 (.306)	.784	.533*** (.516; CI = .322 to .669)
College Gratitude	11–28 (4–28)	23.55 (3.652)	− .569 (.153)	− .243 (.306)	.882	.482*** (.461; CI = .256 to .627)
Total student wellbeing	46–112 (16–112)	80.39 (12.672)	.112 (.153)	− .073 (.306)	.909	.666*** (.642; CI = .483 to .760)

CSSWQ = College Student Subjective Wellbeing Questionnaire; SE = standard error. N = 252; for the retest N = 71

***p ≤ .001

The four identified subscales showed moderate to strong correlations, the coefficients ranging from 0.252 (academic efficacy and college gratitude) to 0.652 (academic satisfaction and academic efficacy), similar to the subscale inter-correlations found in the original CSSWQ study [45] which ranged 0.24 (academic efficacy and school connectedness) to 0.56 (academic efficacy and academic satisfaction). Test–retest Pearson correlations also showed moderate to strong correlations, and the intra-class correlations generally indicated moderate reliability (values between 0.5 and 0.75), with the confidence intervals including some poor (< 0.5) and some good (> 0.75/ < 0.9) values [27] (see Table 2).

Additionally, there was also a trend for males to score higher in all aspects of wellbeing, but none of the differences were statistically significant (all *p*s > 0.1); female/male means (SD) were: academic satisfaction, 18.19 (4.149)/19.01 (4.504); academic efficacy, 17.32 (4.593)/17.95 (5.203); school connectedness, 20.74 (3.796)/21.14 (3.431); college gratitude, 23.50 (3.591)/23.65 (3.799); total student wellbeing, 79.74 (12.196)/81.75 (13.602). The correlations with age were also all non-significant: academic satisfaction, − 0.093; academic efficacy, − 0.025; school connectedness, 0.104; college gratitude, 0.016; total student wellbeing, − 0.006 (all *ps* > 0.1).

Table 3 presents the descriptive statistics for the other questionnaire scales, and for GPA. Each value of skewness ( <|1|) indicated an approximately normal distribution. The alpha coefficients for the SLSS and PANAS positive and negative affect were all good (> 0.8). The alpha coefficients for the big five dimensions in the BFI-10 ranged from 0.294 to 0.672. The relatively low values of alpha may be due to the shortness of the subscales (each included only two items), and these values are similar to those found in previous research [4].

**Table 3:** Descriptive Statistics for SLSS, PANAS, BFI-10, and GPA

	Range (possible)	Mean (SD)	Skewness (SE)	Kurtosis (SE)	Cronbach’s alpha
SLSS	5–30 (5–30)	21.94 (4.327)	− .656 (.153)	1.068 (.306)	.881
*PANAS*					
Positive affect	10–50 (10–50)	32.72 (5.917)	.178 (.154)	.901 (.306)	.846
Negative affect	10–46 (10–50)	23.54 (6.748)	.403 (.154)	− .043 (.306)	.863
*BFI-10*					
Extraversion	2–10 (2–10)	6.38 (1.878)	− .051 (.153)	− .534 (.306)	.672
Agreeableness	3–10 (2–10)	7.33 (1.474)	− .402 (.153)	.353 (.306)	.294
Conscientiousness	2–10 (2–10)	6.40 (1.539)	− .073 (.153)	.031 (.306)	.397
Neuroticism	2–10 (2–10)	5.94 (1.753)	.116 (.153)	− .233 (.306)	.502
Openness	3–10 (2–10)	7.94 (1.615)	− .725 (.153)	.158 (.306)	.469
GPA	1–5 (1–5)	3.37 (.810)	− .499 (.153)	1.184 (.306)	−

SLSS = Students’ Life Satisfaction Scale; PANAS = Positive and Negative Affect Schedule; BFI-10 = 10-Item Big Five Personality Inventory; GPA = grade point average; SE = standard error. N = 252, except for PANAS, N = 251

### Construct validity: nomological network

Pearson correlational analysis indicated that the total CSSWQ-Chinese and all four subscales were moderately/strongly positively correlated with SLSS and with PANAS positive affect, and had small/moderate correlations with BFI-10 extraversion (positive), with PANAS negative affect (negative), and with BFI-10 neuroticism (negative) (see Table 4). These correlations were as expected. Also, BFI-10 agreeableness was positively correlated with academic satisfaction, school connectedness, college gratitude, and the total CSSWQ. BFI-10 conscientiousness was positively correlated with the total scale and each subscale, the strongest correlation being with academic efficacy. BFI-10 openness had small positive correlations with college gratitude and the total CSSWQ score. GPA had moderate positive correlations with academic satisfaction, academic efficacy, and the total CSSWQ score.

**Table 4:** Pearson correlations between the CSSWQ-Chinese and SLSS, PANAS, BFI-10, and GPA

CSSWQ	SLSS	PANAS positive affect	PANAS negative affect	BFI-10 extravert	BFI-10 agreeable	BFI-10 conscientious	BFI-10 neurotic	BFI-10 open	GPA
Academic Satisfaction	.346***	.443***	− .113	.112	.265***	.235***	− .103	.086	.282***
Academic Efficacy	.316***	.500***	− .072	.113	.089	.487***	− .023	.059	.372***
School Connectedness	.467***	.304***	− .264***	.256***	.293***	.166**	− .256***	.106	.090
College Gratitude	.348***	.207***	− .145*	.114	.284***	.175**	− .088	.147*	.051
Total student Wellbeing	.473***	.488***	− .184**	.188**	.290***	.362***	− .143*	.124*	.277***

CSSWQ = College Student Subjective Wellbeing Questionnaire; SLSS = Students’ Life Satisfaction Scale; PANAS = Positive and Negative Affect Schedule; BFI-10 = 10-Item Big Five Personality Inventory; GPA = self-reported grade point average. N = 252, except for PANAS, N = 251

*p ≤ .05; **p ≤ .01; ***p ≤ .001

### Incremental validity

To test if domain-specific student wellbeing has incremental validity in predicting GPA, in comparison with measures of global wellbeing, hierarchical linear regression was undertaken in which step 1 included the PANAS positive and negative affect subscales, the SLSS, and also the Big Five personality dimensions, and gender (females tend to have higher GPA; [46]. This first model showed *R* = 0.326, *R*^*2*^ = 0.106, *F*(9, 241) = 3.186 (*p* = 0.001). The CSSWQ total score was then added in step 2, and this produced a significant change in the model, with *R* = 0.386, *R*^*2*^ = 0.149; change in *R*^*2*^ = 0.043, *p* = 0.001; *F*(10, 240) = 4.212 (*p* < 0.001). Standardised betas for the final model are shown in Table 5. This analysis was independently repeated for each of the four subscales of the CSSWQ. This showed that academic efficacy was the strongest subscale predictor for GPA (*β* = 0.385, p < 0.001), followed by academic satisfaction (*β* = 0.274; p < 0.001), school connectedness (*β* = 0.051; p > 0.1), and college gratitude (*β* = -0.018; p > 0.1).

**Table 5:** Linear regression predicting student grade point average (GPA)

	Standardized beta	t-value
Students’ Life Satisfaction Scale (SLSS)	− .138	− 1.921
Positive and Negative Affect Schedule (PANAS)		
Positive affect	.118	1.561
Negative affect	− .208	− 2.599**
Big Five Inventory (10-item)		
Extraversion	− .064	− 1.002
Agreeableness	− .104	− 1.590
Conscientiousness	.065	.988
Neuroticism	.137	1.822
Openness	.083	1.359
Gender	− .154	− 2.518*
College Student Subjective Wellbeing Questionnaire (CSSWQ), total score	.272	3.483***

Gender was coded as 0 for female and 1 for male. N = 251

*p ≤ .05; **p ≤ .01; ***p ≤ .001

### Demographic variables

The distributions of responses for exercise (skewness = − 0.026), and money received monthly from family (skewness = 0.640) were approximately normal, while for smoking (skewness = 4.682) and alcohol drinking (skewness = 1.292), the distributions were strongly positively skewed. 55% (n = 139) of the participants had never drank, and 45% (n = 113) had drank before at different frequencies. Over 90% (n = 229) of the participants had never smoked before (only 23 participants had).

Pearson correlational analysis was conducted between the CSSWQ-Chinese (including the subscales) and demographic questions. The results are shown in Table 6. In general, there was little correlation between each aspect of students’ wellbeing and each of the other variables. Exercise had a small positive correlation with academic efficacy. In this study, drinking had weak correlations with all aspects of wellbeing, while smoking had a small positive correlation with school connectedness. Money had small negative correlations with academic satisfaction and academic efficacy.

**Table 6:** Pearson Correlations between the CSSWQ-Chinese and Demographics

	Exercise	Drink alcohol	Smoke	Monthly money received from family
Academic satisfaction	.063	.028	− .015	− .132*
Academic efficacy	.135*	− .027	− .032	− .120
School connectedness	− .021	.081	.130*	.030
College gratitude	.048	− .068	.058	.035
Total Student wellbeing	.080	.003	.037	− .071

CSSWQ = College Student Subjective Wellbeing Questionnaire; N = 252, except for Money, N = 250. *p ≤ .05

## Discussion

The aim of the current study was to develop and validate a Chinese version of the College Student Subjective Wellbeing Questionnaire (CSSWQ). Although the results of the exploratory factor analysis had some degree of ambiguity, the four-factor structure was the most clear and logical. The loadings in the pattern matrix showed a structure that was identical with the original English CSSWQ [44]. The four CSSWQ subscales showed moderate/strong inter-correlations, as also found with the English CSSWQ [44, 45].

The means for each aspect of the CSSWQ-Chinese were above the midpoint of the ranges, indicating relatively high levels of subjective wellbeing. However, in general, the students in the current study had lower levels of wellbeing in all aspects compared to American college students in the original revised CSSWQ study. The means of academic satisfaction (AS = 18.45 ± 4.275), academic efficacy (AE = 17.52 ± 4.797), college gratitude (CG = 23.55 ± 3.652), school connectedness (SC = 20.87 ± 3.680), and total student wellbeing (TSW = 80.39 ± 12.672) were all smaller than the counterparts in the original study (AS = 21.65 ± 4.84; AE = 23.04 ± 3.88; CG = 25.80 ± 2.45; SC = 22.27 ± 4.09; TSW = 92.76 ± 12.24) [44]. Scores of college gratitude ranked the highest in the subscales in both countries. These results are consistent with the wellbeing of the two countries’ general population. According to the World Happiness Report 2019, the overall happiness of mainland Chinese ranked 93 out of the 156 countries in the list, while the overall happiness of Americans ranked 19 out of 156 [19]. Among the factors considered to measure the happiness level of a nation (e. g., individual life evaluation, positive affect, negative affect), China was reported to have higher levels of happiness evaluation and social support, but lower levels of happiness equity and freedom of choice compared to America [19].

In the revised English CSSWQ study [44], approximately normal distributions were shown in most subscales (skewness and kurtosis <|2|), except for the college gratitude subscale which had a skewness of − 2.23 and a kurtosis of 9.80. In the current study, college gratitude also had the highest value of skewness (− 0.569), although within an acceptable range. Cronbach’s alpha coefficients for the total CSSWQ-Chinese score and the scores of each of its subscales met the generally accepted minimal value of > 0.7, and were largely consistent with the original study [44], indicating adequate to strong internal consistency. Test–retest Pearson correlations showed moderate to strong correlations between the two tests, while the intra-class correlations generally indicated moderate reliability. Wellbeing dimensions may be expected to remain relatively stable, at least over relatively short intervals. Among all the aspects of the CSSWQ-Chinese, academic satisfaction scores were relatively the most stable with a test–retest Pearson correlation of 0.746, while college gratitude scores were relatively the least stable, with a test–retest Pearson correlation of 0.482. It is possible that these aspects of wellbeing could change over time while somebody is at university, perhaps following certain experiences (e. g., college gratitude may increase after receiving good exam results, although so may academic satisfaction and efficacy). Therefore, more research needs to be done on the stability of, and influences on, the different aspects of college student subjective wellbeing.

The construct validity of the CSSWQ was supported by correlations between the CSSWQ-Chinese and other scales in the nomological network, which were in line with previous research findings for the CSSWQ, e. g., positive with PANAS positive affect and with life satisfaction, and negative with PANAS negative affect [44], with similar observed coefficient sizes. The CSSWQ was also positively correlated with extraversion, and negatively correlated with neuroticism, consistent with general measures of wellbeing [31]. The CSSWQ also showed positive correlations with agreeableness, conscientiousness, and (more weakly) with openness. The observed pattern of correlations between the CSSWQ and big five dimensions in the current research shows much consistency with a recent study in the USA [56].

Assumed as a correlate of college students’ wellbeing, self-reported GPA in this study was found to have moderate positive correlations with academic efficacy, academic satisfaction, and the total CSSWQ score. Comparing with the revised English CSSWQ study [44], the Chinese/English bivariate correlation coefficients for the CSSWQ and GPA were: academic satisfaction 0.282/0.50,academic efficacy 0.372/0.48; college gratitude 0.090/0.17; school connectedness 0.051/0.11; total student wellbeing 0.277/0.42. Although the correlations in the current study were all smaller than those in the original study, it is consistent that GPA was a much stronger correlate of academic satisfaction, academic efficacy, and total student wellbeing than for college gratitude and school connectedness. Evidence was also found to support the CSSWQ-Chinese’s incremental validity, as it (especially the subscale of academic efficacy) was a significant predictor of GPA, after controlling for other aspects of wellbeing and other predictors of academic achievement.

The current study also investigated associations between college student wellbeing and drinking alcohol, smoking, exercise, and monthly family subsidy. It was found that drinking alcohol had little correlation with all aspects of students’ wellbeing, which may be due to the balanced-out effect of academic side effects and social benefits of drinking. On one hand, it is well supported by the literature that (excessive) alcohol drinking is related to poorer physical and mental wellbeing of individuals [5, 13, 34], and poorer academic performance of college students, primarily because drinking takes time away from studying (e. g., missing class due to hangovers) [39]. On the other hand, drinking, as long as below a certain amount, may help enhance a person’s positive emotions, and ability to cope with negative emotions; for college students, drinking often serves as a way for relaxing, celebrating, and sharing happy/sad feelings with friends [8].

Second, smoking had a small positive correlation with school connectedness, while it may be expected to relate to higher risk of psychological problems and poorer student wellbeing, based on the evidence of previous research [14, 17]. However, similar to social drinking, smoking might serve a socializing function among students and enhance their social relationships and subjective connectedness to the college society [33].

Third, exercise had a small positive correlation with academic efficacy, which is consistent with an existing longitudinal study which found that regular exercise relates to better mental wellbeing [49].

Last, monthly family subsidy had small negative correlations with academic satisfaction and academic efficacy. It might be the case that college students with more extra disposable money have less academic motivation, or, financial assistance may be a reflection of a family’s parenting style, which could directly affect their children’s financial coping behaviours, and indirectly affect their wellbeing [50]. However, as the current research was conducted at a joint-venture university with relatively high tuition fees, students at this university may generally have financial backgrounds much above the nation’s average level. The trend that an increased amount of money is related to poorer academic satisfaction and efficacy may not apply to students in most other Chinese universities. Future research may investigate this.

### Limitations and further research

The study is limited by the use of a convenience sample, and with a cross-sectional design the study cannot show any causal relationships between the variables. Also, volunteer bias may potentially have influenced the sample characteristics and study results, e. g., the overall wellbeing from this study may be better than the population level because more outgoing and helpful individuals, with higher wellbeing, may have been more willing to participate in the research. Also, there were limitations with the questionnaires chosen to validate the CSSWQ. For example, the BFI-10 only has two items for each personality dimension, so it may be better to use a longer scale (e.g., the BFI-44), which may also allow for facet-level analysis. Additionally, more data could be collected for construct validity by comparing with other relevant scales, such as the Satisfaction with Life Scale [9], the Adult Hope Scale [51], and the Beck Anxiety Inventory [2], which were used by Renshaw [44]. Also, future studies could use alternative data collection methods such as online questionnaires which can deploy the survey more rapidly, and can ensure the completeness of each survey [18]. Also, research with larger and more diverse samples may further test the structure of the Chinese CSSWQ using confirmatory factor analysis, and also test for the single higher-order factor of covitality (general student wellbeing), as identified by Renshaw and Bolognino [45] and Renshaw [44].

Further research may also explore how disposable money or economic dependence affect college students’ wellbeing in China, investigate cross-cultural comparisons of college students’ wellbeing, and further test the stability of wellbeing traits over time, by measuring wellbeing at different time points throughout the school year. Other components of college student subjective wellbeing (in addition to the four assessed by the CSSWQ) may also be investigated.

## Conclusion

The current study developed a Chinese translation of the College Student Subjective Wellbeing Scale (CSSWQ), and analysis of data for this scale showed evidence for validity and reliability. The Chinese CSSWQ could be useful for future research to (1) monitor students’ wellbeing in Chinese universities, (2) explore influences on student wellbeing, and (3) measure student wellbeing as an outcome in intervention studies. While there is a lot of depression among college students, there has been a relative lack of data on other aspects of the psychological wellbeing of this group, and a relative lack of instruments that specifically measure college students’ wellbeing in China. The use of the Chinese CSSWQ could help gather more data about students' wellbeing, and be useful in research to promote the wellbeing and happiness of Chinese university students.

## Data Availability

The dataset supporting the conclusions of this article is included within the article (and its additional file(s)).

## Abbreviations

CSSWQ: College Student Subjective Wellbeing Questionnaire
GPA: Grade point average
DALY: Disability adjusted life year
SLSS: The Students' Life Satisfaction Scale
PANAS: The Positive and Negative Affect Schedule
BFI-10: The 10-Item Big Five Personality Inventory
EFA: Exploratory factor analysis
